# Electrodeposition of porous graphene networks on nickel foams as supercapacitor electrodes with high capacitance and remarkable cyclic stability

**DOI:** 10.1186/1556-276X-9-672

**Published:** 2014-12-12

**Authors:** Shaolin Yang, Bingchen Deng, Ruijing Ge, Li Zhang, Hong Wang, Zihan Zhang, Wei Zhu, Guanzhong Wang

**Affiliations:** Hefei National Laboratory for Physical Sciences at Microscale, and Department of Physics, University of Science and Technology of China, Hefei, Anhui 230026 People’s Republic of China; School of the Gifted Young, University of Science and Technology of China, Hefei, Anhui 230026 People’s Republic of China

**Keywords:** Graphene, Porous, Electrodeposition, Nickel foam, Crumpled morphology, Supercapacitor, Electrode

## Abstract

**Abstract:**

We describe a facile, low-cost, and green method to fabricate porous graphene networks/nickel foam (PG/NF) electrodes by electrochemical deposition of graphene sheets on nickel foams (NFs) for the application of supercapacitor electrodes. The electrodeposition process was accomplished by electrochemical reduction of graphene oxide (GO) in its aqueous suspension. The resultant binder-free PG/NF electrodes exhibited excellent double-layer capacitive performance with a high rate capability and a high specific capacitance of 183.2 mF cm^-2^ at the current density of 1 mA cm^-2^. Moreover, the specific capacitance maintains nearly 100% over 10,000 charge-discharge cycles, demonstrating a remarkable cyclic stability of these porous supercapacitor electrodes.

**PACS:**

82.47.Uv (Electrochemical capacitors); 82.45.Fk (Electrodes electrochemistry); 81.05.Rm (Fabrication of porous materials)

**Electronic supplementary material:**

The online version of this article (doi:10.1186/1556-276X-9-672) contains supplementary material, which is available to authorized users.

## Background

Supercapacitors have attracted tremendous attention due to their novel characteristics, including rapid charging-discharging rate, high power density, long cycle life, and high dynamic of charge propagation [1–4]. Supercapacitors generally fall into two categories according to the specific energy storage mechanism [5, 6]. One type is called electrical double-layer capacitors (EDLCs) which store energy with the pure electrostatic charge adsorbed at the electrode-electrolyte interface. The second type is the pseudocapacitors that store energy through fast and reversible Faradic surface or near-surface redox reactions. Compared with pseudocapacitors, EDLCs usually have higher rate performance, better cycling stability, and longer lifetime [7]. Carbon materials including active carbon, carbon nanotubes, xerogel, mesoporous carbon, and carbide-derived carbon have been developed as electrodes in EDLCs [5, 8–13].

In the last few years, graphene, a two-dimensional carbon material, has been extensively studied as a candidate for EDLC electrode material owing to its large specific surface area, very high electrical conductivity, and profuse interlayer structure in comparison with traditional porous carbon materials [6, 7, 14–16]. However, graphene-based electrodes often suffer from stacking and self-aggregation of graphene sheets due to the strong π-π interaction among graphene layers, which will impede the diffusion of electrolyte, decrease the accessible surface area, and consequently reduce the effective capacitance of the electrodes [17, 18]. To overcome this problem, three-dimensional (3D) graphene architectures have been developed as supercapacitor electrodes, which endow the graphene-based electrodes with ultra-large accessible surface area, interconnected conductive network, and thereby better capacitive performance [19–24]. On the other hand, the contact resistance between 3D graphene and the metal current collector is another limitation for supercapacitor performance [25]. In this regard, strategies have been developed by two groups to fabricate binder-free supercapacitor electrodes by depositing porous graphene networks directly into pores of nickel foams (NFs) [25, 26]. The 3D scaffold structure of NFs endows the graphene-based electrodes with large surface area and short diffusion length of ions, while the binder-free deposition enhances the internal binding between graphene materials and the NF current collectors, and thus results in low contact resistance. Shi et al. fabricated graphene hydrogel/NF electrodes through immersing NFs filled with graphene oxide (GO) suspension in vitamin C solution overnight followed by heating at 60°C for 2 h [26]. Feng et al. prepared graphene aerogel/NF electrodes by freeze-drying of GO hydrogel-NF precursor and subsequently thermal annealing [25]. Nevertheless, these procedures are complicate and time-consuming, especially high temperature is needed for the latter one.

Herein, we report a facile, environment-friendly, and easily controllable method of fabricating supercapacitor electrodes by electrodeposition of porous graphene networks onto the scaffolds of nickel foams (PG/NFs) through electrochemical reduction of a concentrated graphene oxide suspension. Electrodeposition has been proved to be an effective and green way to fabricate graphene-based electrodes, since the procedure was accomplished by electrochemical route without involvement of high temperature, toxic reactants, and binder material as well as additional transfer process [27–29]. The PG/NF electrodes showed excellent double-layer capacitive performance with high specific capacitance, extraordinary cyclic stability, and high rate capability. These results can be attributed to the following reasons: large specific surface area resulted from the porous structure of PF/NF electrodes and crumpled surface morphology of the deposited graphene sheets; low contact resistance and ultra-long durability caused by the compact binder-free contact between graphene materials and the NF current collectors; and good conductivity of the graphene materials and short diffusion distance for ions from porous graphene networks to NF current collectors. These advantageous features endow the PG/NF electrodes with a potential application of supercapacitors with very high stability and energy capacity.

## Methods

### Materials

Natural graphite flake (-325 mesh, 99.8%) was bought from Alfa Aesar, Ward Hill, MA, USA, and used for producing graphene oxide. Lithium perchlorate trihydrate (LiClO_4_ · 3H_2_O; 99%), sulfuric acid (H_2_SO_4_; 98%), hydrochloric acid (HCl; 36%), potassium permanganate (KMnO_4_; 99.5%), potassium hydroxide (KOH; 85%), and sodium nitrate (NaNO_3_; 99%) were purchased from Sinopharm Chemical Reagent Co., Ltd., Shanghai, China, and used as received.

### Preparation of PG/NFs

Graphene oxide was prepared by the oxidation of natural graphite flake according to Hummers’ method and preserved in water under continuous stirring [30]. The electrodeposition of graphene sheets onto nickel foam employed a three-electrode system consisting nickel foam with the thickness of 1 mm and pore density of 110 ppi as working electrode, Pt wire as counter electrode and Ag/AgCl as reference electrode. The electrolyte was the mixed solution of 0.15 M LiClO_4_ and 7.5 mg mL^-1^ graphene oxide suspension previously ultra-sonicated for 60 min. In a typical procedure, NFs previously immersed in the electrolyte were subjected to mild sonication for 15 min to ensure that the electrolyte diffuses into their pores. After that, the electrodepositions were conducted under constant potentials of -1.0, -1.1, -1.2, and -1.3 V with deposition times of 300, 400, 500, 600, 700, and 800 s. The electrodeposited PG/NF electrodes were then immersed in deionized water to remove the unwanted GO and LiClO_4_. To further increase the conductivity of the as-deposited graphene sheets, the electrodes were electrochemically reduced at 6 M KOH solution with a cyclic voltammetry scan with a potential range from 0 to -1.5 V at a rate of 50 mV s^-1^ for ten cycles. The mass of the deposited graphene materials is about 1.0 to 2.0 mg, obtained from weight difference of the sample before and after electrochemical deposition.

### Characterization

The PG/NFs were freeze-dried and used for further characterization. Scanning electron microscopy (SEM) was performed on a field emission scanning electron microscope (JSM-6700 F, JEOL, Tokyo, Japan), Raman spectra were recorded on a micro-Raman microscope (LabRAM HR-800, Horiba Jobin Yvon, Lille, France) with 633-nm laser, X-ray photoelectron spectroscopy (XPS) was performed on an ESCALAB 250Xi X-ray Photoelectron spectrometer (Thermo Fisher Scientific Inc., Waltham, MA, USA), and X-ray diffraction (XRD) analysis was carried out in a Rigaku RINT-TTR III X-ray diffractometer (Rigaku Corporation, Tokyo, Japan). The specific surface area of PG/NF electrodes was measured by methylene blue adsorption (see details in Additional file 1).

### Electrochemical measurements

Electrochemical tests were conducted in the electrolyte of 6 M KOH solution using CHI760E electrochemical workstation (Chenhua Instruments Co., Ltd., Shanghai, China) with the same three-electrode system as it was for previous electrodeposition. Cyclic voltammetry (CV) scans were performed with a potential range from -0.2 to -1.0 V at scan rates ranging from 10 to 1,000 mV s^-1^. Galvanostatic charge-discharge curves were also recorded between -0.2 and -1.0 V. Electrochemical impedance spectroscopy (EIS) tests were conducted with the frequency ranging from 0.02 Hz to 100 kHz and the amplitude being 5 mV.

## Results and discussion

**Figure 1:**
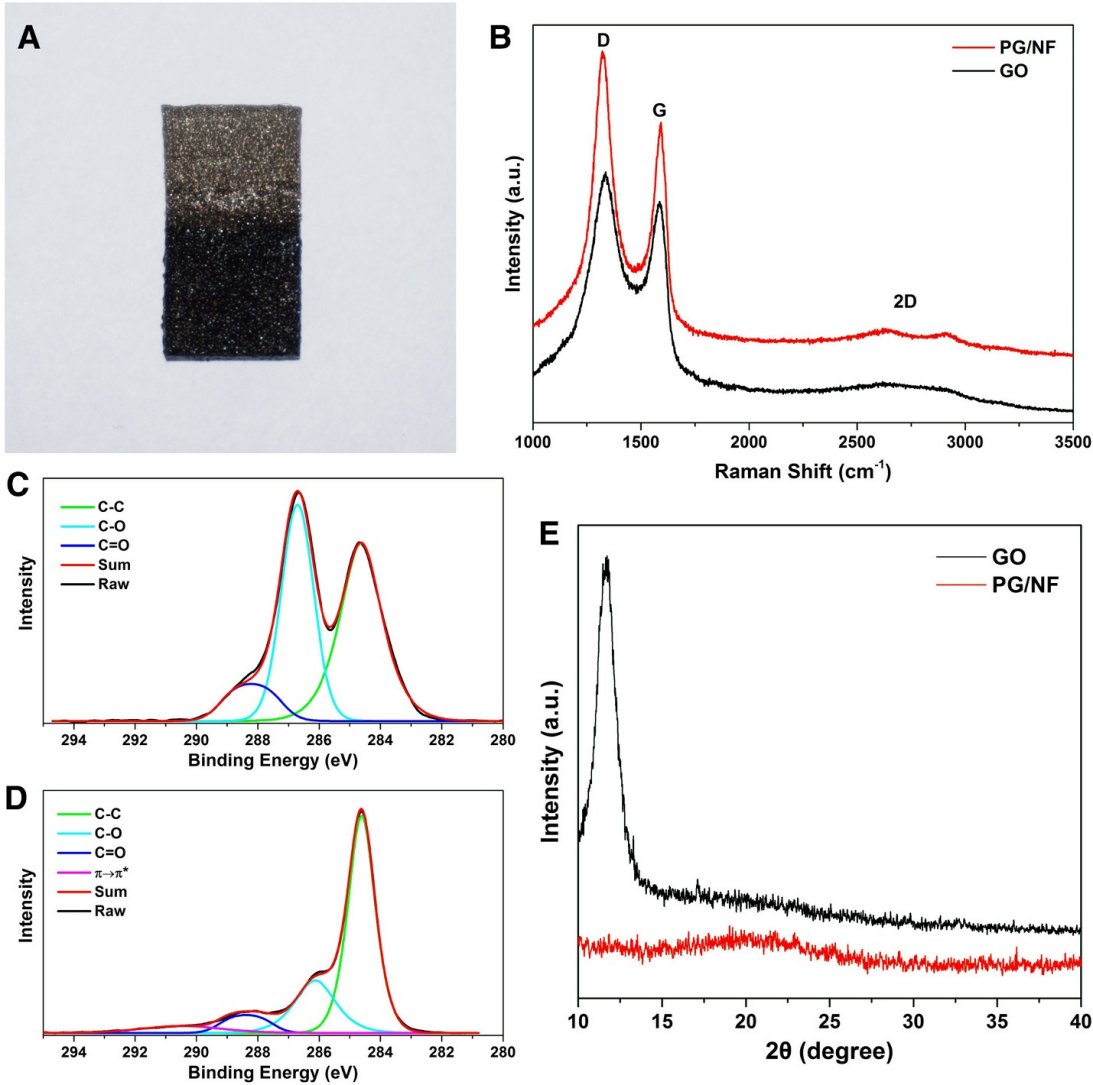
**Characterization of GO and PG/NF. (A)** Photograph of a piece of PG/NF, in which the graphene sheet coated part is in black color. **(B)** Raman spectra of GO and PG/NF. **(C, D)** C 1 s XPS spectra for (C) GO and (D) PG/NF. **(E)** XRD patterns of GO and PG/NF.

The fabrication process of PG/NF electrodes was accomplished by electrochemical route without involvement of high temperature, toxic reactants and additional transfer process, representing a quick, green, low-cost, and easily controllable approach to fabricating graphene-based supercapacitor electrodes. As shown in Figure 1A, the optical photograph of the as-prepared PG/NF clearly shows that black graphene materials were coated on the NF.

The Raman spectra of GO and PG/NF (Figure 1B) show two strong G and D bands, which could be attributed to carbon sp^2^ domains and structural defects, respectively [31]. The G band of GO shifted towards that of pristine graphite (from 1,585 to 1,592 cm^-1^) for the PG/NF electrode, suggesting the reduction of GO. The intensity ratio of D/G bands for PG/NF is 1.45, higher than that of GO (1.1). This increase implies a decrease in average size of the graphitic domains upon the electrochemical reduction, which can be ascribed to the formation of large amounts of smaller new conjugated domains [32]. Moreover, a broadened 2D band near 2,700 cm^-1^ was found in the spectrum of PG/NF only, which is regarded as an evident band of graphene resulted from a two-phonon resonant scattering process [33].

Figure 1C shows the C 1 s XPS of GO, which can be decomposed into three peaks at 284.6, 286.7, and 288.2 eV, corresponding to C-C, C-O, and C = O bonds, respectively [34]. In the spectra of PG/NF (Figure 1D), the intensity of C-O (286.1 eV) and C = O (288.4 eV) peaks reduces significantly, while the C-C (284.6 eV) peak becomes more prominent. These results suggest that most oxygen-containing functional groups of GO were successfully removed by the electrodeposition and the successive electrochemical reduction process. Furthermore, a π → π* shake-up satellite peak appeared at 290.5 eV for XPS spectra of PG/NF, which is a characteristic of aromatic or conjugated systems [35].

XRD patterns of GO and PG/NF are presented in Figure 1E. A strong diffraction peak is observed for GO at 11.7°, corresponding to an interlayer spacing distance of 0.756 nm. This distance is significantly larger than that of pristine graphite (0.335 nm), probably due to the intercalation of oxygen-containing functional groups in GO sheets [36]. Instead of GO peak, a broad band appeared at about 20° for PG/NF sample, suggesting that the interlayer spacing of PG/NF had reduced to 0.44 nm probably by virtue of the removal of the functional groups. Furthermore, the broadened and weakened peak of PG/NF could be ascribed to the poor ordering of the deposited graphene sheets along their stacking direction [37, 38].

**Figure 2:**
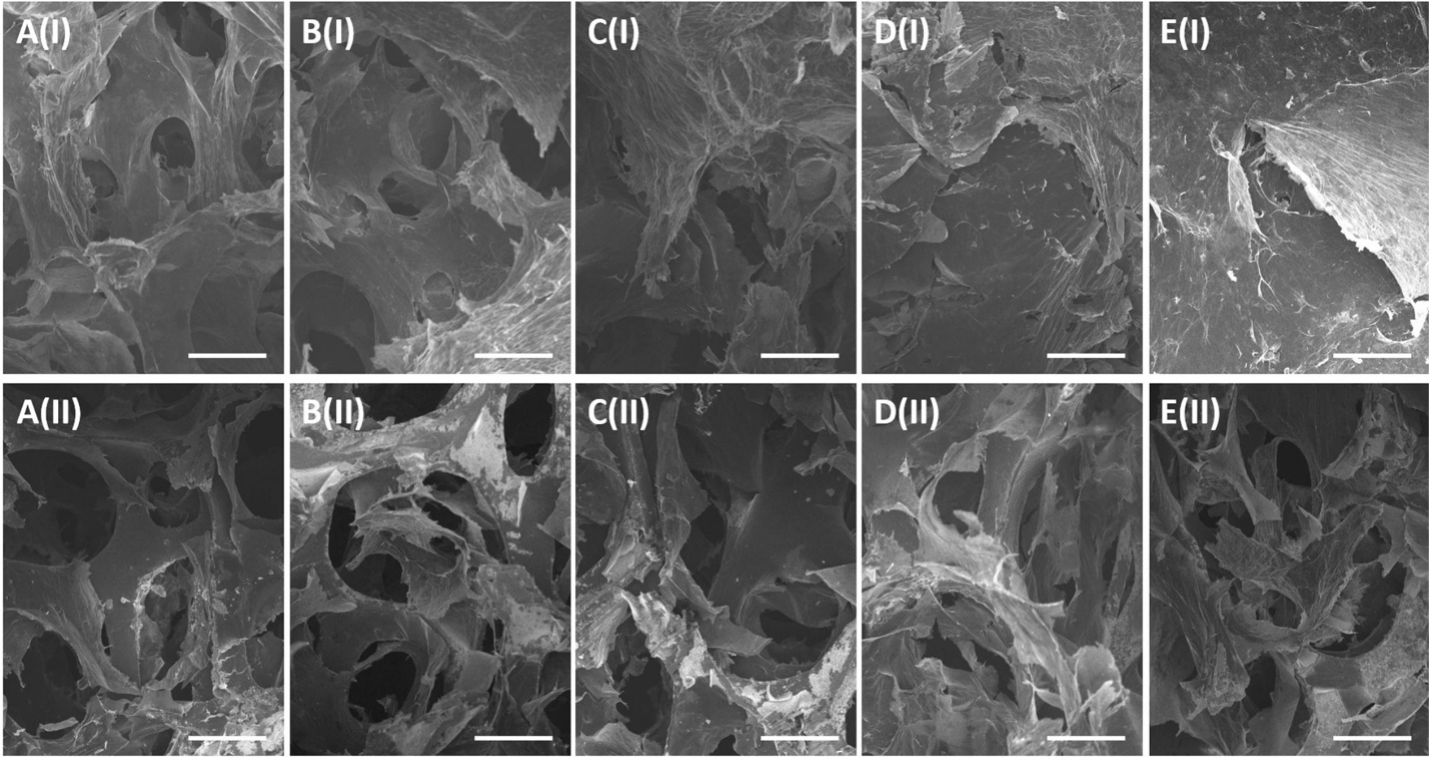
**SEM images of PG/NFs deposited under -1.2 V with different deposition times. (A)** 300, **(B)** 400, **(C)** 500, **(D)** 600, and **(E)** 700 s. A(I) to E(I) are the top views, and A(II) to E(II) are the cross-sections. Scale bar: 100 μm.

**Figure 3:**
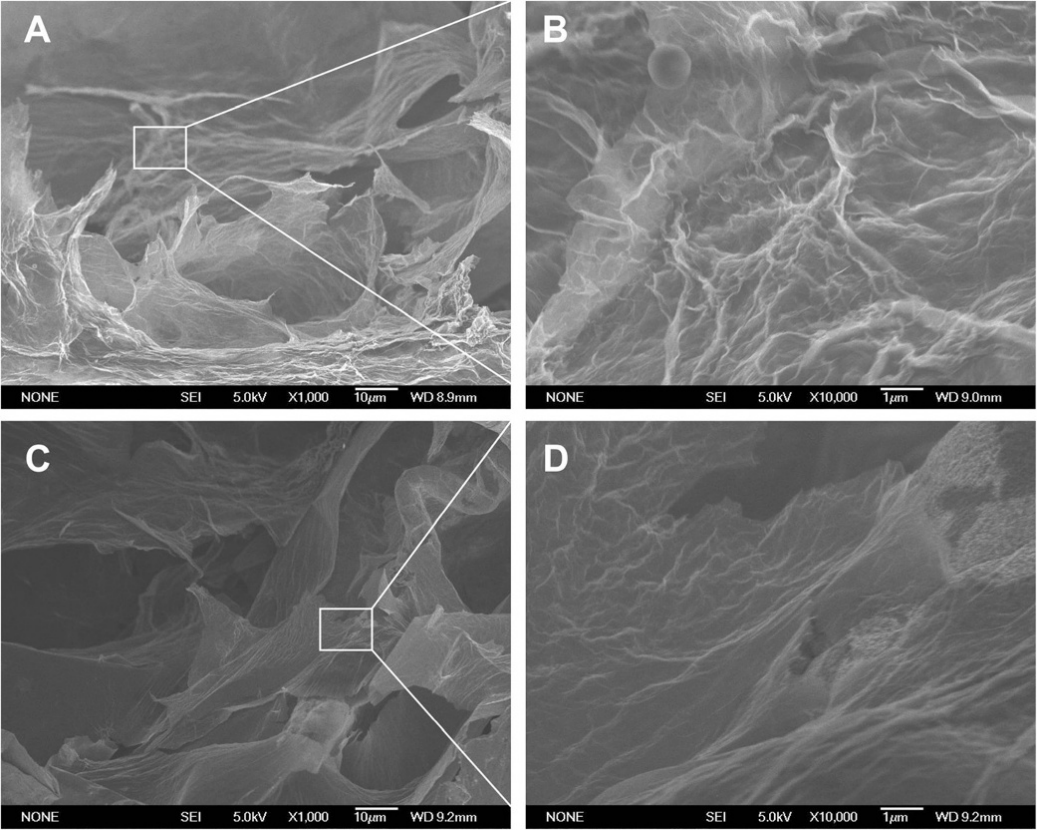
**SEM images of PG/NFs at high magnifications. (A)** and **(B)** are the top views, and **(C)** and **(D)** are the cross-sections. **(B)** and **(D)** are the enlarged views of the squares in **(A)** and **(C)**, respectively.

Electrodepositions of graphene sheets were firstly conducted at the potential of -1.2 V with deposition times of 300, 400, 500, 600, and 700 s. The mass of deposited graphene materials per unit area of PG/NF increased from 1.0 to 1.3, 1.5, 1.8, or 2.0 mg cm^-2^ as the deposition time increased from 300 to 400, 500, 600, or 700 s. Figure 2 displays the SEM images of top view and cross-section of PG/NF electrodes. Compared with bare NF shown in Additional file 1: Figure S1, the PG/NF SEM images show that the graphene materials have been deposited onto the outsides and insides of the pores of the NFs. As shown from Figure 2A(I) and A(II) to Figure 2E(I) and E(II), with the increase of deposition time, more graphene sheets were deposited onto the NF frameworks. Notably, more graphene sheets were coated on the outside of the NFs, which can be seen from the top view SEM images shown from Figure 2A(I) to E(I). For electrodeposition processes longer than 600 s, the deposited graphene sheets began to cover the pores of the NF electrodes, which would block the diffusion of electrolytes into those pores. Figure 3 shows the high magnification SEM images of the PG/NFs. Figure 3B,D is the enlarged views of the squares in Figure 3A,C, respectively. It is obvious that the deposited graphene sheets formed porous structure with pore sizes in tens of micrometers from both top view and cross-section view. In addition, crumpled morphology is also observed on the surface of the porous graphene networks (Figure 3B,D). These porous architectures and crumpled surface topography could enlarge the surface area and thus enhance the specific capacitance of the electrodes.

**Figure 4:**
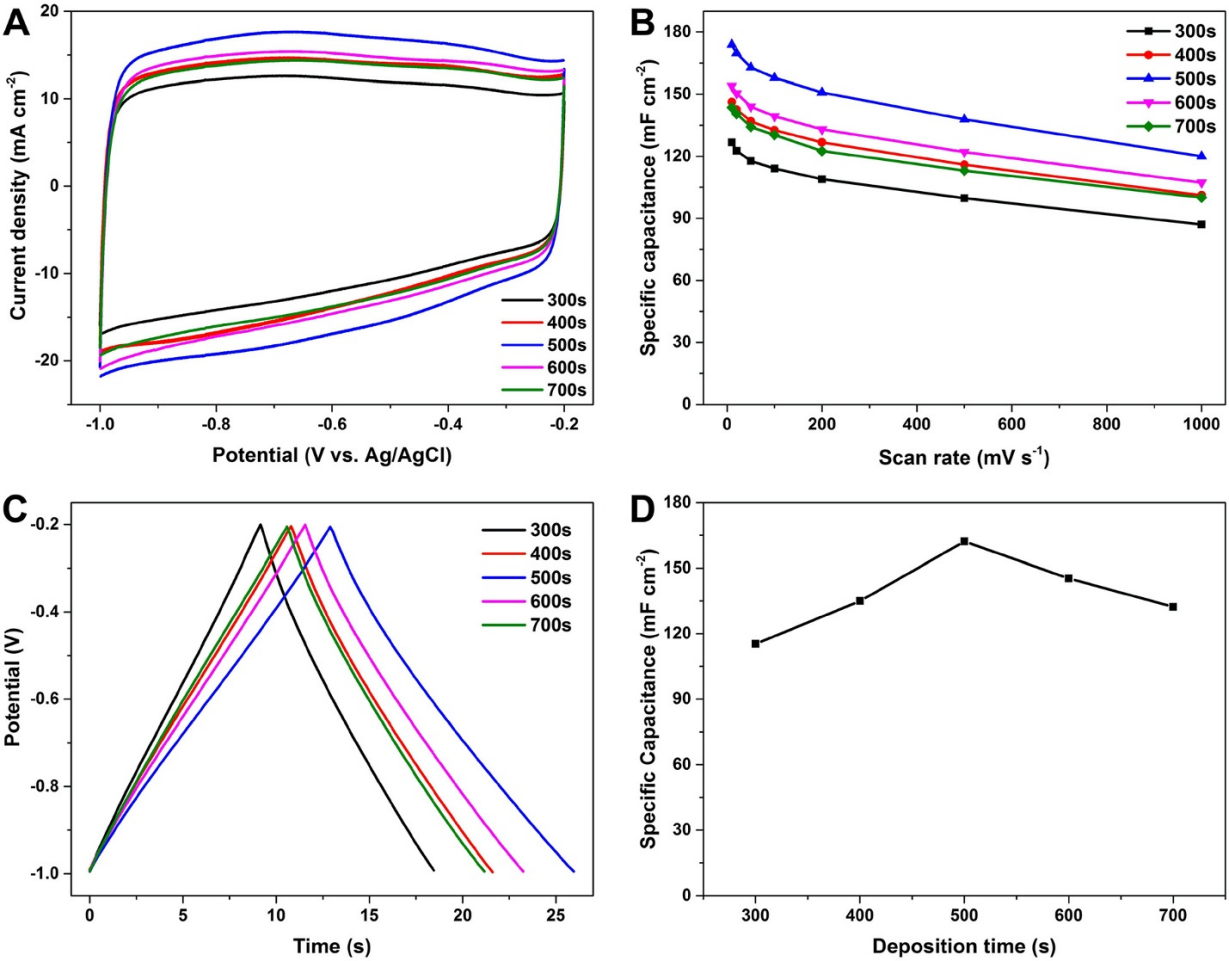
**Electrochemical measurements of the PG/NF deposited at the potential of -1.2 V with different deposition times. (A)** Cyclic voltammetry curves at the scan rate of 100 mV s^-1^ and **(B)** plots of specific capacitances versus scan rate. **(C)** Galvanostatic charge-discharge curves and **(D)** plot of specific capacitances at current density of 10 mA cm^-2^.

The electrochemical tests of the PG/NFs deposited under the potential of -1.2 V with deposition times of 300, 400, 500, 600, and 700 s were shown in Figure 4. Considering the incompatibility between the weight of the deposited graphene materials (1.0 ~ 2.0 mg) and the NF supports (60 ~ 70 mg), the specific capacitance of the PG/NFs in area units were used as measurements instead of the mass-specific capacitance. Figure 4A compares the CV curves of these PG/NF electrodes at the potential scan rate of 100 mV s^-1^. All CV curves are in a nearly perfect rectangular shape, indicating pure electric double-layer capacitances and rapid charge propagations at the electrode/electrolyte interfaces [26]. Figure 4B displays the comparison of the specific capacitances of these electrodes versus scan rate from 10 to 1,000 mV s^-1^. These specific capacitances decrease with the increase of scan rate, probably due to an increase of ion diffusion-related resistance [39]. It is obvious that the specific capacitances of the PG/NF electrodes do not always increase with the deposition time. They increase with deposition time from 300 to 500 s because of larger surface area generated by larger amounts of graphene materials deposited. However, the specific capacitances began to decrease with the deposition time of 600 s and continued to reduce for longer deposition. This phenomenon can be explained by that the covering of the pores of the NFs by excessive graphene materials as shown in Figure 2D(I) and E(I), impeding ion dispersion into the pores of the PG/NF electrodes and thereby decreasing the accessible surface area. This can be verified by the specific surface area measured by methylene blue adsorption to be 0.913, 1.026, 1.268, 1.122, and 1.010 m^2^ cm^-2^ for PG/NF electrodes fabricated with the deposition time of 300, 400, 500, 600, and 700 s, respectively.

Figure 4C shows the galvanostatic charge-discharge curves of these PG/NF electrodes at a current density of 10 mA cm^-2^. All curves exhibit a symmetric triangle shape without voltage drop observed at the onset of discharge, reflecting a very good double-layer capacitive behavior with a fast I-V response as well as relatively low equivalent series resistance of these electrodes [39]. The specific capacitances calculated from these charge-discharge curves are shown in Figure 4D. At the current density of 10 mA cm^-2^, the specific capacitance of the PG/NF deposited with the deposition time of 300 s was 115.3 mF cm^-2^. It increased to 135.0 mF cm^-2^ for the deposition time of 400 s, achieved the maximum of 162.2 mF cm^-2^ for 500 s, and decreased to 145.3 mF cm^-2^ for 600 s, and to 132.3 mF cm^-2^ for 700 s. This variation regularity of specific capacitance with the increase of deposition time agrees well with the results obtained from the CV tests.

**Figure 5:**
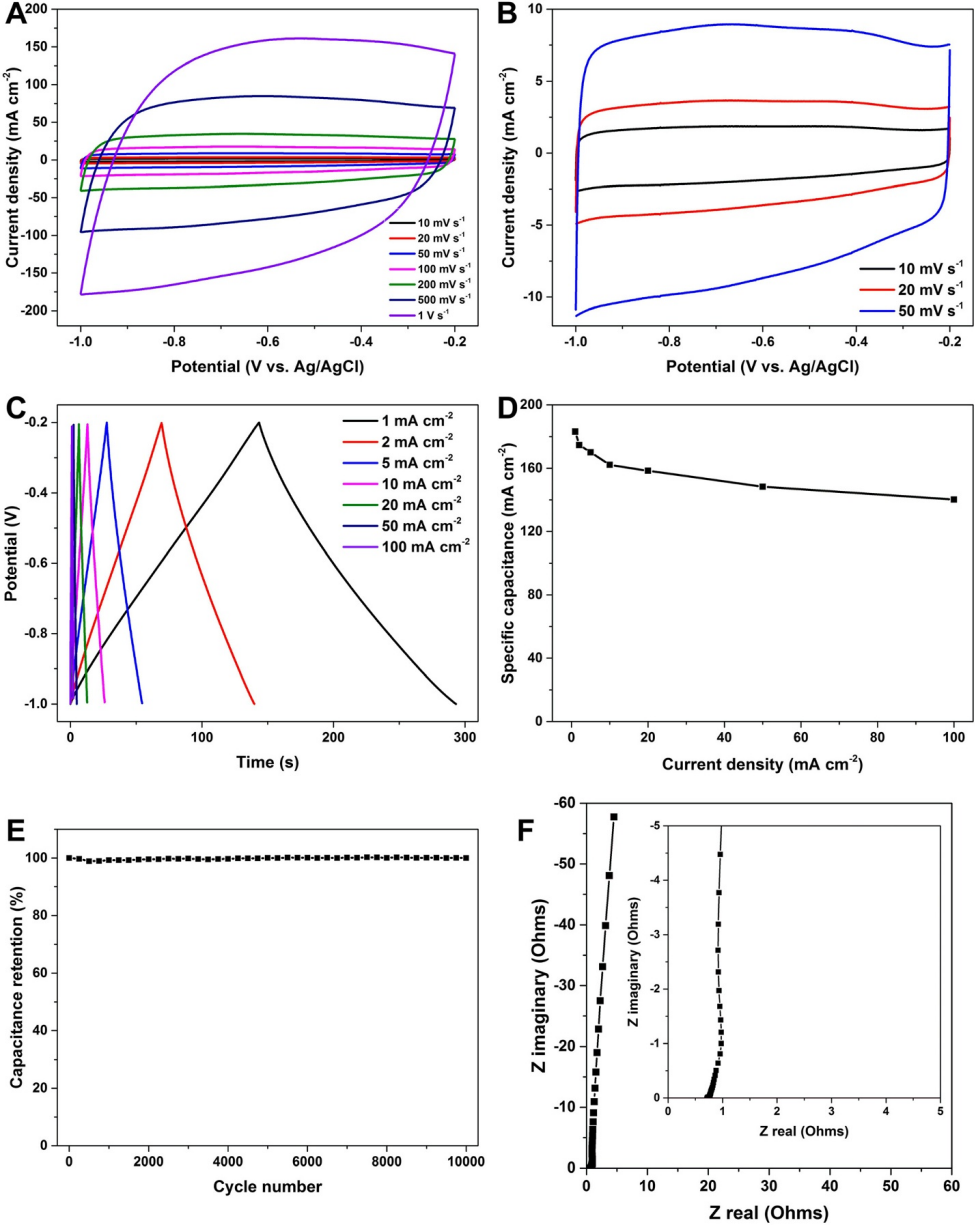
**Electrochemical measurements of the PG/NF deposited under the potential of -1.2 V with the deposition time of 500 s. (A)** Cyclic voltammetry curves at different scan rates. **(B)** Magnification of the CV curves for the scan rates of 10, 20, and 50 mV s^-1^ in (A). **(C)** Galvanostatic charge-discharge curves measured with different current densities. **(D)** Plot of specific capacitances at different current densities. **(E)** Cyclic stability test at scan rate of 500 mV s^-1^. **(F)** Nyquist plot of EIS. Inset: plot on enlarged scale.

In the following experiment, we performed detailed electrochemical characterization of the PG/NF electrode prepared under the deposition potential of -1.2 V with the time of 500 s. Figure 5A presents the CV behavior of the PG/NF under different scan rates from 10 to 1,000 mV s^-1^, and Figure 5B shows the CV curves for the scan rates from 10 to 50 mV s^-1^ on enlarged scale. The rectangular shape and the absence of redox peaks for all curves imply that the charge dispersion at the electrode surfaces follows the mechanism of electric double-layer capacitors [28]. The CV curve keeps a quasi-rectangular shape even at a high rate of 1 V s^-1^, suggesting that the PG/NF electrode has a good rate performance and a low internal resistance [40].

As shown in Figure 5C, the charge-discharge curves under different current densities between 1 and 100 mA cm^-2^ exhibit a very good triangle shape, indicating an almost ideal double-layer capacitive behavior of this electrode. In addition, only 0.14 V of voltage drop at the beginning of discharge is observed even at the current density of 100 mA cm^-2^, reflecting a low internal resistance of the PG/NF electrode. The calculated specific capacitances under different current densities are shown in Figure 5D. The highest specific capacitance of 183.2 mF cm^-2^ was obtained at the current density of 1 mA cm^-2^. The specific capacitance was still 140.3 mF cm^-2^ (76.6% of that measured at 1 mA cm^-2^) as the current density rose high as 100 mA cm^-2^, suggesting a good rate performance of the PG/NF electrode. The specific capacitance of our PG/NF (183.2 mF cm^-2^ at the current density of 1 mA cm^-2^) is higher than those of most previously reported high-rate carbon-based EDLCs such as graphene hydrogels deposited NF (45.6 mF cm^-2^ at the current density of 0.67 mA cm^-2^), [26] laser-scribed graphene film (3.67 mF cm^-2^ at the current density of 1 mA cm^-2^) [41], and onion-like carbon (1.7 mF cm^-2^ at the scan rate of 1 V s^-1^) [42]. This could be attributed to the high conductivity and crumpled surface topography of the deposited graphene sheets as well as porous structure of the PF/NF electrodes.

The cyclic stability of the PG/NF electrode was evaluated by repeating CV scan from -0.2 to -1.0 V at a scan rate of 500 mV s^-1^. As shown in Figure 5E, the specific capacitance maintains nearly unchanged over 10,000 cycles, demonstrating a remarkable electrochemical stability of our PG/NF electrode. This extraordinary cycling stability probably be attributed to the 3D porous structure of the electrode, good electrochemical stability of the porous graphene networks, and the good contact between deposited graphene sheets and the NF current collector.

The rapid ion transport inside the PG/NF electrode was further confirmed by electrochemical impedance spectroscopy. As shown in Figure 5F, the Nyquist plot of the PG/NF electrode begins with a short 45° region, attributing to the porous structure of the electrode [26]. The nearly vertical line at the low frequencies on the Nyquist plot reflects a pure capacitor behavior of the electrode. The equivalent series resistance value of the PG/NF obtained from the intersection of the Nyquist plot with the real axis is 0.8 Ω. In addition, there is no semicircular region observed in the Nyquist plot at high frequencies, suggesting pure double-layer capacitive behavior of the PG/NF electrode.

**Figure 6:**
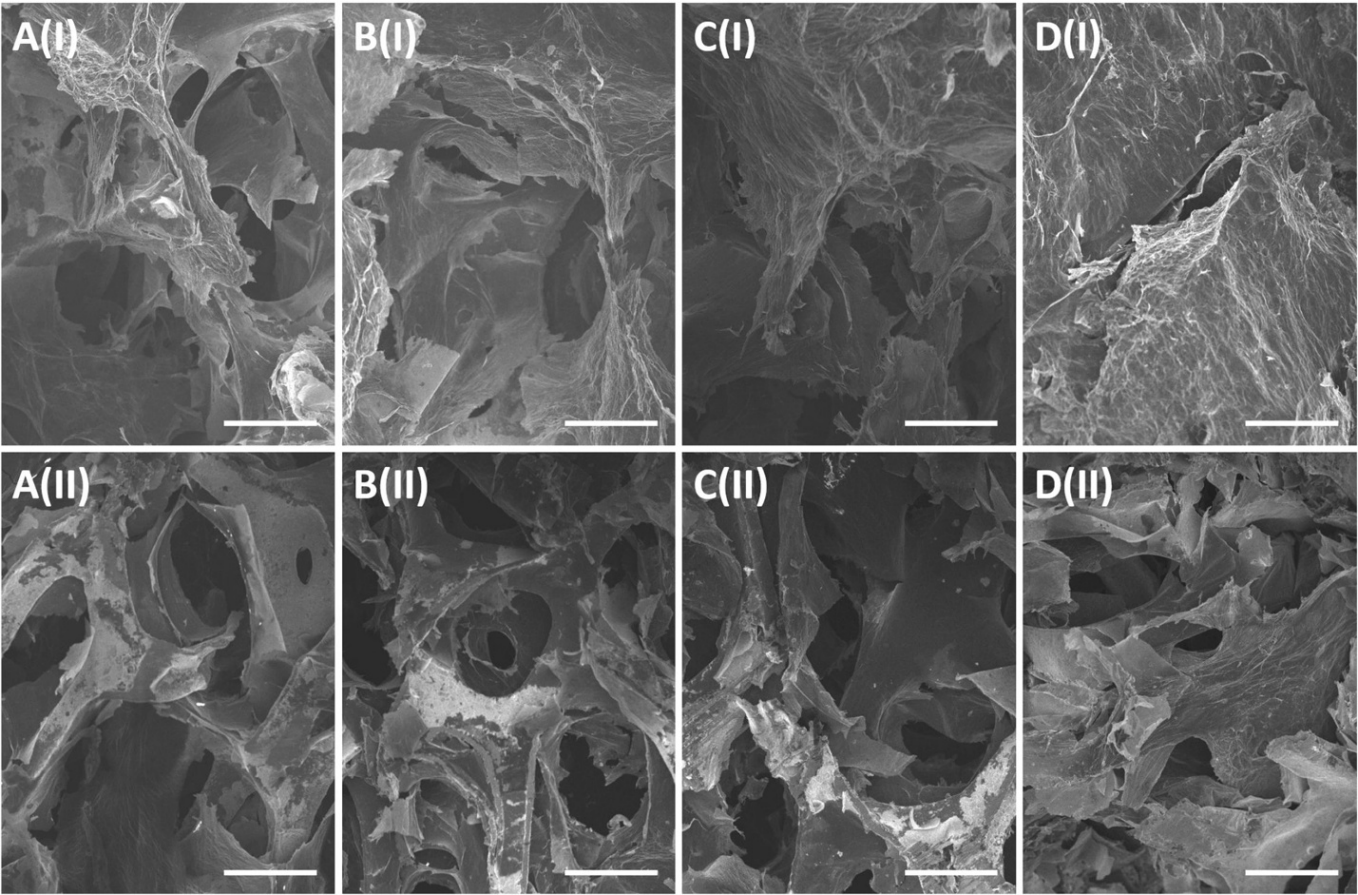
**SEM images of the PG/NFs deposited with the time of 500 s under different potentials. (A)** -1.0, **(B)** -1.1, **(C)** -1.2, and **(D)** -1.3 V. A(I) to D(I) are the top views, and A(II) to D(II) are the cross-sections. Scale bar: 100 μm.

**Figure 7:**
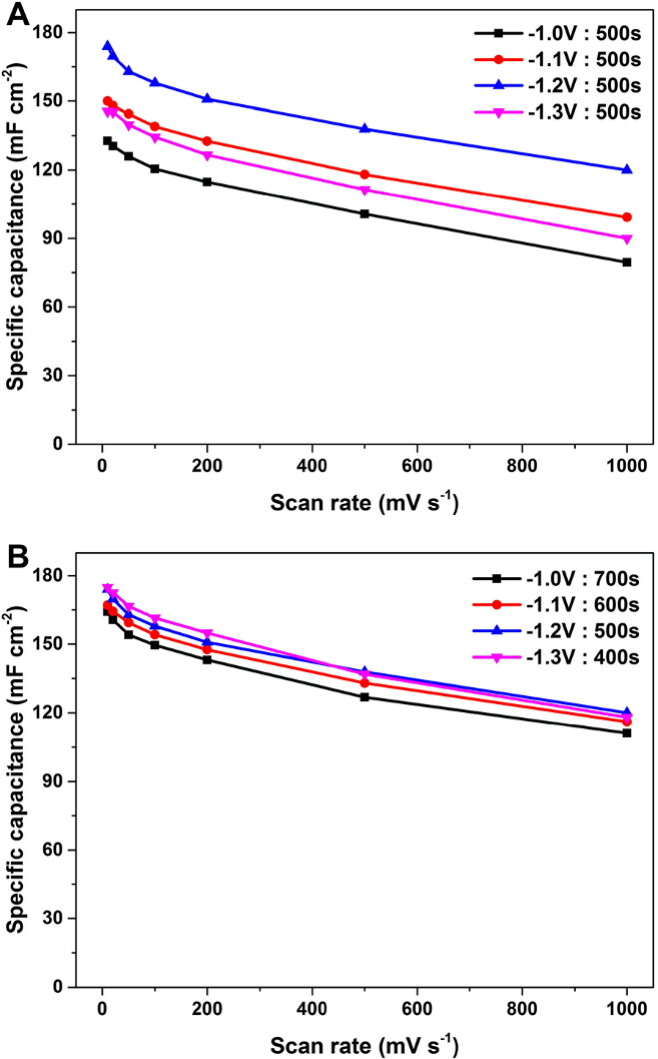
**Plots of specific capacitances for PG/NFs deposited under different potentials with different times. (A)** Plots of specific capacitances for PG/NF electrodes deposited under different potentials with the deposition time of 500 s versus scan rate. **(B)** Plots of specific capacitances versus scan rate for PG/NF electrodes deposited under constant potentials and deposition times of -1.0 V and 700 s, -1.1 V and 600 s, -1.2 V and 500 s, and -1.3 V and 400 s.

We also performed a systematic investigation of the influence of deposition potential on the specific capacitance of the PG/NF electrodes. Additional file 1: Figure S2 displays the linear sweep voltammogram of nickel foam in GO deposition electrolyte scanned from 0 to -1.5 V, from which can be observed the deposition current density increase nearly linear with the decreasing potential below -0.6 V. This is because more negative deposition potential would produce stronger electric field and thereby larger deposition current. Figure 6 shows the SEM images of the PG/NFs deposited with the deposition time of 500 s under potentials of -1.0, -1.1, -1.2, and -1.3 V. As shown in the top view and cross-section SEM images, the amount of deposited graphene materials increases with more negative deposition potential. It is suggested that more negative deposition potential produced larger deposition current and resulted in more graphene sheets deposited on the NFs. However, the pores of the NF were finally blocked by excessive graphene under the deposition potential of -1.3 V, which is similar to what had happened at -1.2 V with the deposition time of 600 or 700 s. As shown in Figure 7A, the specific capacitances increased with the deposition potential from -1.0 to -1.2 V but began to decrease when the potential reached -1.3 V. The variation of the capacitance for different potentials should be attributed to the consequences of the varied deposition potential on the surface areas and accessibility to the insides of the electrodes, which is similar to that of the varied deposition time at a constant potential of -1.2 V.

We also studied the PG/NFs prepared under the potentials of -1.0, -1.1, and -1.3 V with different deposition times. As shown in Additional file 1: Figure S3, S4, and S5, under each deposition potential, the amount of graphene materials for PG/NFs increases with the deposition time and the pores of the NFs are finally obstructed for an enough long deposition time. The comparison of specific capacitances of these electrodes prepared under different deposition potentials and times versus scan rate from 10 to 1,000 mV s^-1^ is displayed in Additional file 1: Figure S6. The capacitances for the PG/NF electrodes deposited under each potential firstly increased with the increasing deposition times but ultimately decreased. The largest specific capacitances obtained were met at the deposition time of 700 s for -1.0 V, 600 s for -1.1 V, and 400 s for -1.3 V, respectively. Figure 7B presents the comparison of the specific capacitances versus scan rate for these PG/NF electrodes deposited under constant potentials and deposition times of -1.0 V and 700 s, -1.1 V and 600 s, -1.2 V and 500 s, and -1.3 V and 400 s. There are no obvious differences among the specific capacitances of these electrodes. These results suggest that deposition potential would mainly influence deposition rate and determine the amount of graphene sheets on the electrode with deposition time together. The specific capacitance is mainly affected by the accessible surface area of the graphene sheets deposited on the electrode.

## Conclusions

In summary, we demonstrate a feasible, green, and low-cost route to fabricate supercapacitor electrodes by electrodeposition of porous graphene networks on nickel foams. The resultant porous graphene networks/nickel foam electrodes exhibited excellent double-layer capacitive performance with a high rate capability, remarkable cycle stability, and high specific capacitance of 183.2 mF cm^-2^ at the current density of 1 mA cm^-2^. The excellent supercapacitive performance can be ascribed to crumpled surface morphology and high conductivity and electrochemical stability of the deposited graphene sheets, the 3D porous structure of the PG/NF electrodes, short ion diffusion distance inside the electrodes, and good contact of graphene materials and the NF current collectors. After a systematic study of the specific capacitances of the PG/NF electrodes deposited under different potentials with different deposition times, we found that deposition potential mainly influences the deposition rate and a longer deposition time is needed to achieve the maximum capacitance under more positive potential.

## Electronic supplementary material

Additional file 1: **Supporting method and supporting figures.** Supporting method: measurement of the specific surface areas of PG/NF electrodes. Supporting figures: Figure S1: SEM image of bare nickel foam. Figure S2: Linear sweep voltammogram of nickel foam in 7.5 mg mL^-1^ GO and 0.15 M LiClO_4_ solution at the scan rate of 50 mV s^-1^. Figure S3: SEM images of top view of the PG/NFs deposited under the potential of -1.0 V with deposition times of (A) 500, (B) 600, (C) 700, and (D) 800 s. Figure S4: SEM images of top view of the PG/NFs deposited under the potential of -1.1 V with deposition times of (A) 500, (B) 600, and (C) 700 s. Figure S5: SEM images of top view of the PG/NFs deposited under the potential of -1.3 V with deposition times of (A) 300, (B) 400, and (C) 500 s. Figure S6: Plots of specific capacitance of the electrodes prepared under deposition potentials of (A) -1.0, (B) -1.1, and (C) -1.3V with different deposition times versus scan rate. (DOC 2 MB)

## Abbreviations

NF: nickel foam
PG/NF: porous graphene networks/nickel foam
EDLCs: electrical double-layer capacitors
3D: three-dimensional
CVD: chemical vapor deposition
SEM: scanning electron microscope
XPS: X-ray photoelectron spectroscopy
XRD: X-ray diffraction
CV: cyclic voltammetry
EIS: electrochemical impedance spectroscopy.
