# Supplementary material for: Electrodeposition of porous graphene networks on nickel foams as supercapacitor electrodes with high capacitance and remarkable cyclic stability
Source: Nanoscale Res Lett. 2014 Dec 12;9:672. doi: 10.1186/1556-276X-9-672 (PMC4493843; doi:10.1186/1556-276X-9-672)
Supplement: Supplementary file 1 — Additional file 1: Supporting method and supporting figures. Supporting method: measurement of the specific surface areas of PG/NF electrodes. Supporting figures: Figure S1: SEM image of bare nickel foam. Figure S2: Linear sweep voltammogram of nickel foam in 7.5 mg mL-1 GO and 0.15 M LiClO4 solution at the scan rate of 50 mV s-1. Figure S3: SEM images of top view of the PG/NFs deposited under the potential of -1.0 V with deposition times of (A) 500, (B) 600, (C) 700, and (D) 800 s. Figure S4: SEM images of top view of the PG/NFs deposited under the potential of -1.1 V with deposition times of (A) 500, (B) 600, and (C) 700 s. Figure S5: SEM images of top view of the PG/NFs deposited under the potential of -1.3 V with deposition times of (A) 300, (B) 400, and (C) 500 s. Figure S6: Plots of specific capacitance of the electrodes prepared under deposition potentials of (A) -1.0, (B) -1.1, and (C) -1.3V with different deposition times versus scan rate. (DOC 2 MB) [file 11671_2014_2496_MOESM1_ESM.doc]

**Additional file for**

Electrodeposition of Porous Graphene Networks on Nickel Foams as Supercapacitor Electrodes with High Capacitance and Remarkable Cyclic Stability

*Shaolin Yang^a^, Bingchen Deng^b^, Ruijing Ge^b^, Li Zhang^a^, Hong Wang^a^, Zihan Zhang^a^, Wei Zhu^a^,* and Guanzhong Wang^a^,**

^a^ Hefei National Laboratory for Physical Sciences at Microscale, and Department of Physics, University of Science and Technology of China, Hefei, Anhui, 230026, P. R. China

^b^ School of the Gifted Young, University of Science and Technology of China, Hefei, Anhui, 230026, P. R. China

1. **Supporting Method**

**Measurement of the specific surface areas of PG /NF electrodes**: A PG/NF electrode with size of 2 cm^2^ was placed into 6 mL aqueous solution containg methylene blue (MB) with concentration of 200 mg L^-1^. After the adsorption reached equilibrium in 24 h, the concentration of MB was determined by UV-vis spectroscopy. The mass of adsorbed MB was calculated by *M*_MB_ = (*C*_o_ – *C*_e_)*V*, where *C*_o_ and *C*_e_ are respectively the initial and equilibrium concentrations of MB solution (mg L^-1^), *V* is the volume of dye solution (L). Since the surface area covered by 1 mg of adsorbed MB is a constant (2.54 m^2^), the specific surface area (*S*_S_) of PG/NF can be calculated by adsorption of MB with the following equation:

*S*_S_ (m^2^ cm^-2^) *=* 2.54×*M*_MB_/*A*

A is the area of PG/NF electrode, here A=2.

1. **Supporting Figures**


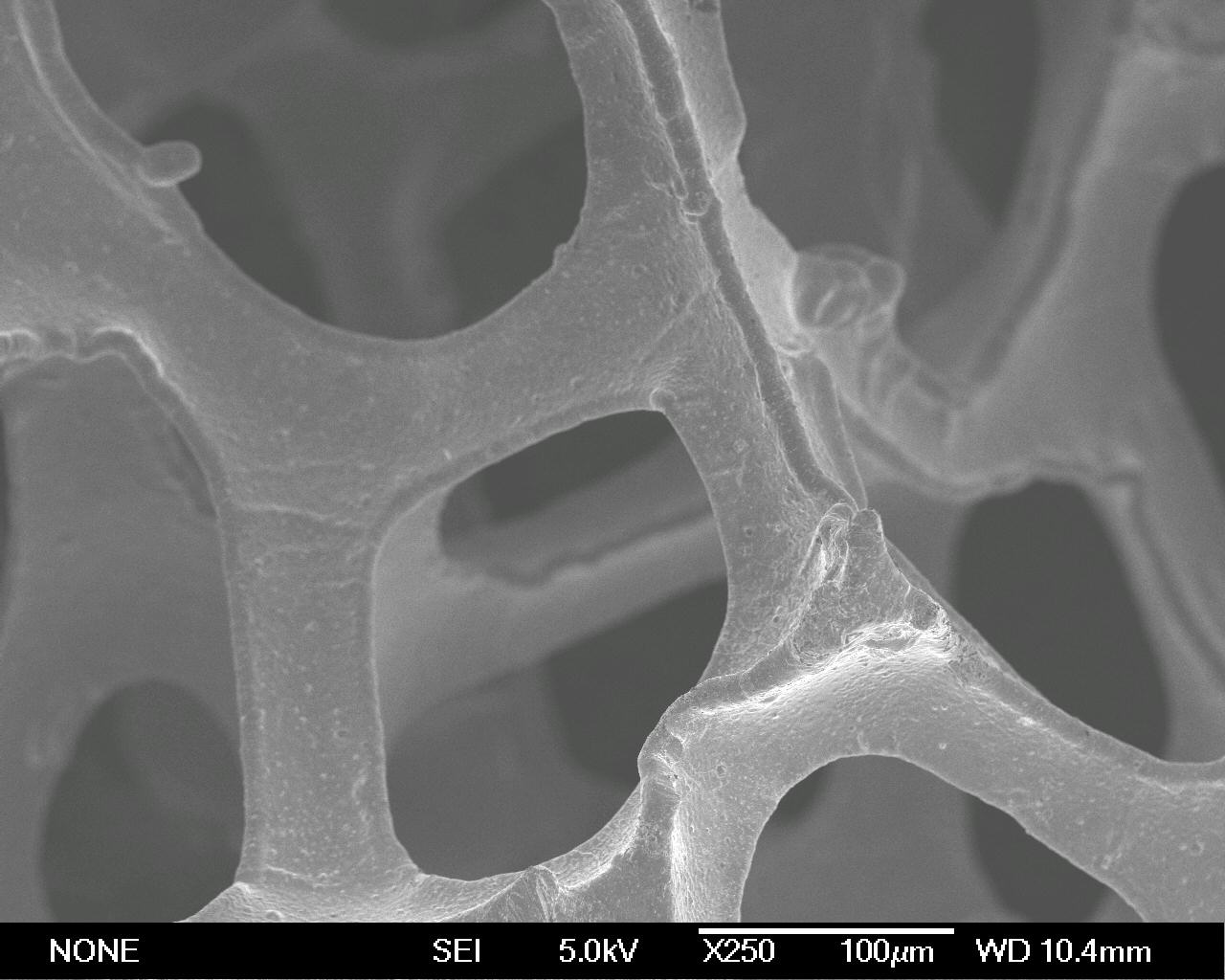


**Figure S1** SEM image of bare nickel foam.


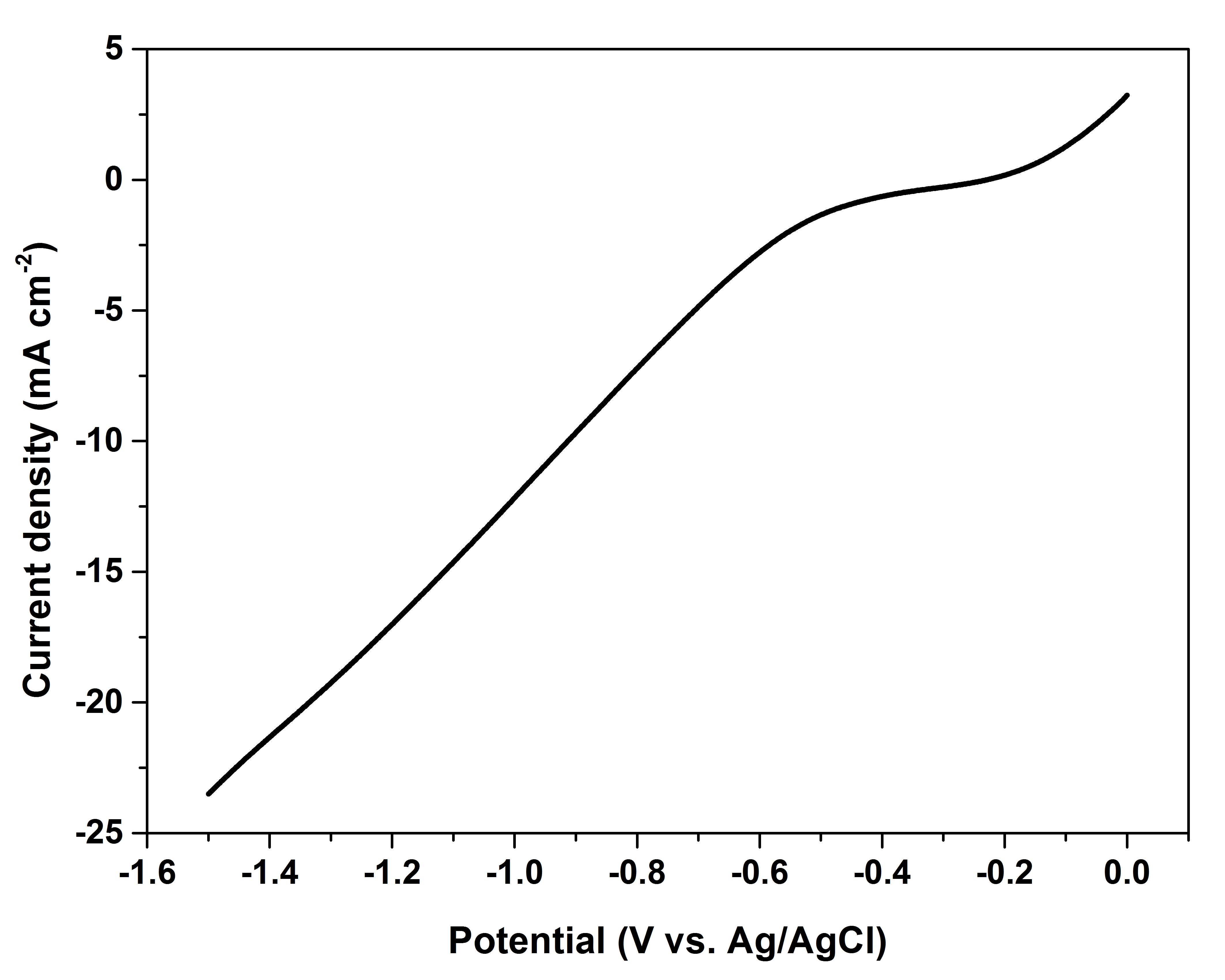


**Figure S2** Linear sweep voltammogram of nickel foam in 7.5 mg mL^-1^ GO and 0.15 M LiClO_4_ solution at the scan rate of 50 mV s^-1^.


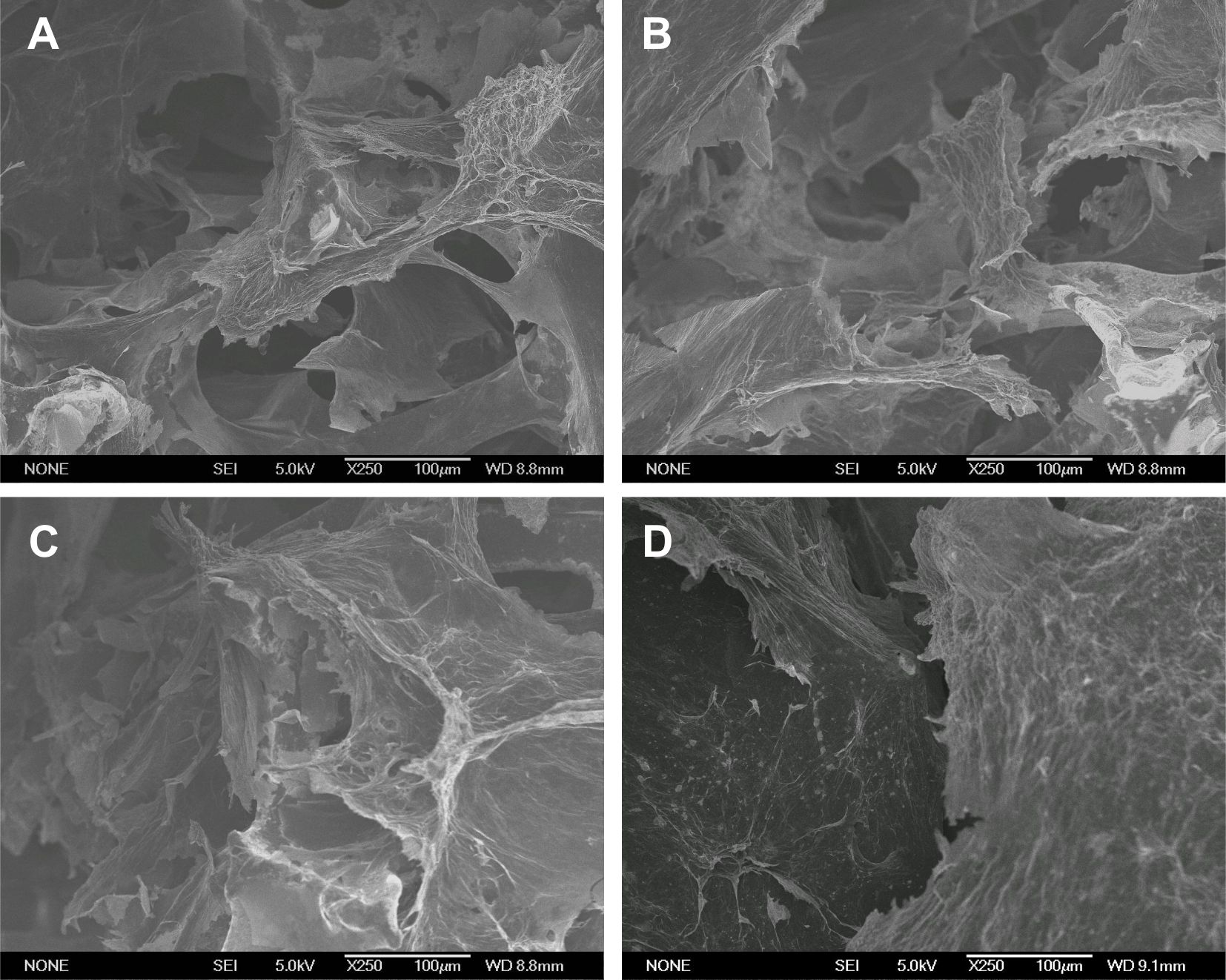


**Figure S3** SEM images of top view of the PG/NFs deposited under the potential of -1.0 V with deposition times of (A) 500, (B) 600, (C) 700, and (D) 800 s.


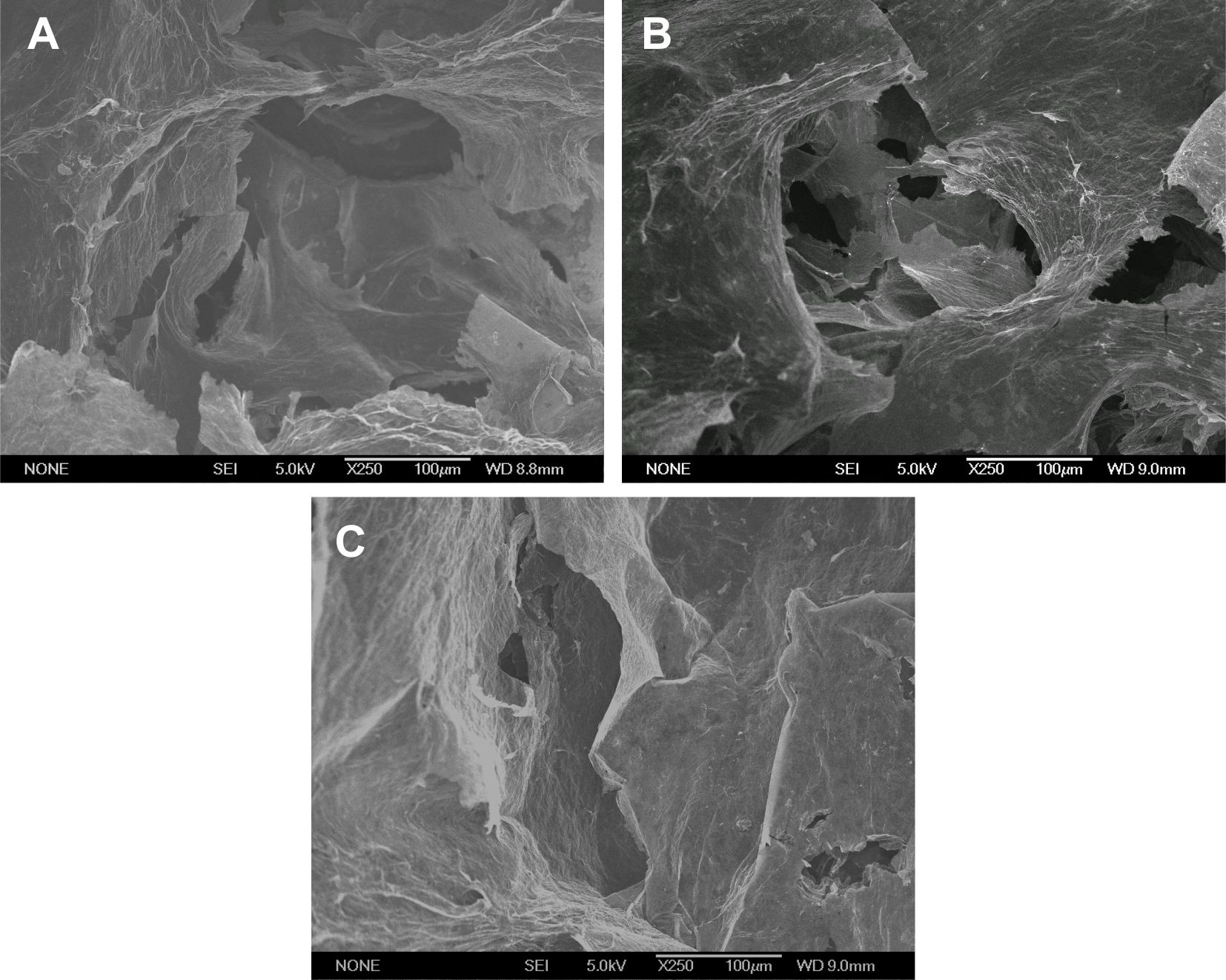


**Figure S4** SEM images of top view of the PG/NFs deposited under the potential of -1.1 V with deposition times of (A) 500, (B) 600, and (C) 700 s.


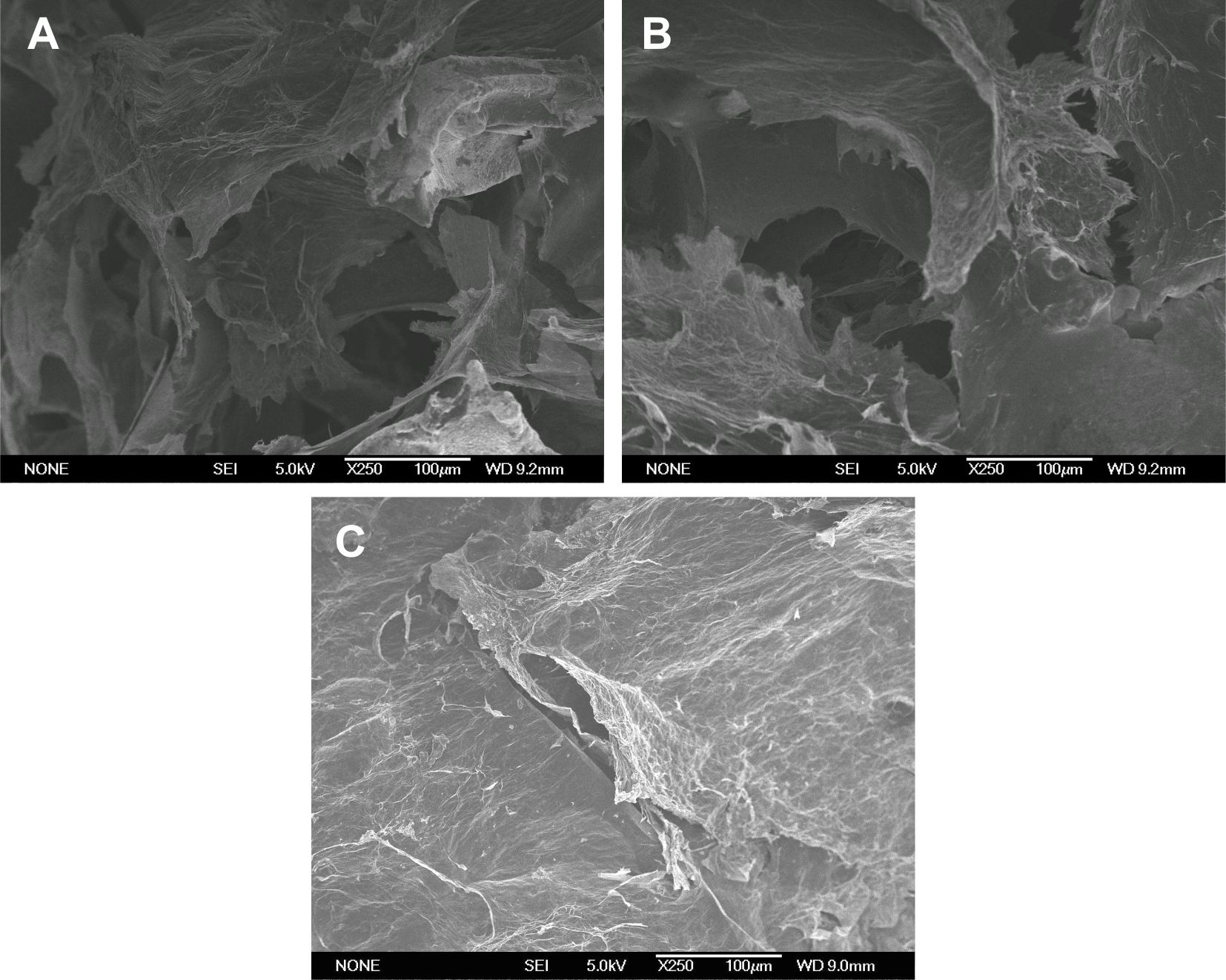


**Figure S5** SEM images of top view of the PG/NFs deposited under the potential of -1.3 V with deposition times of (A) 300, (B) 400, and (C) 500 s.


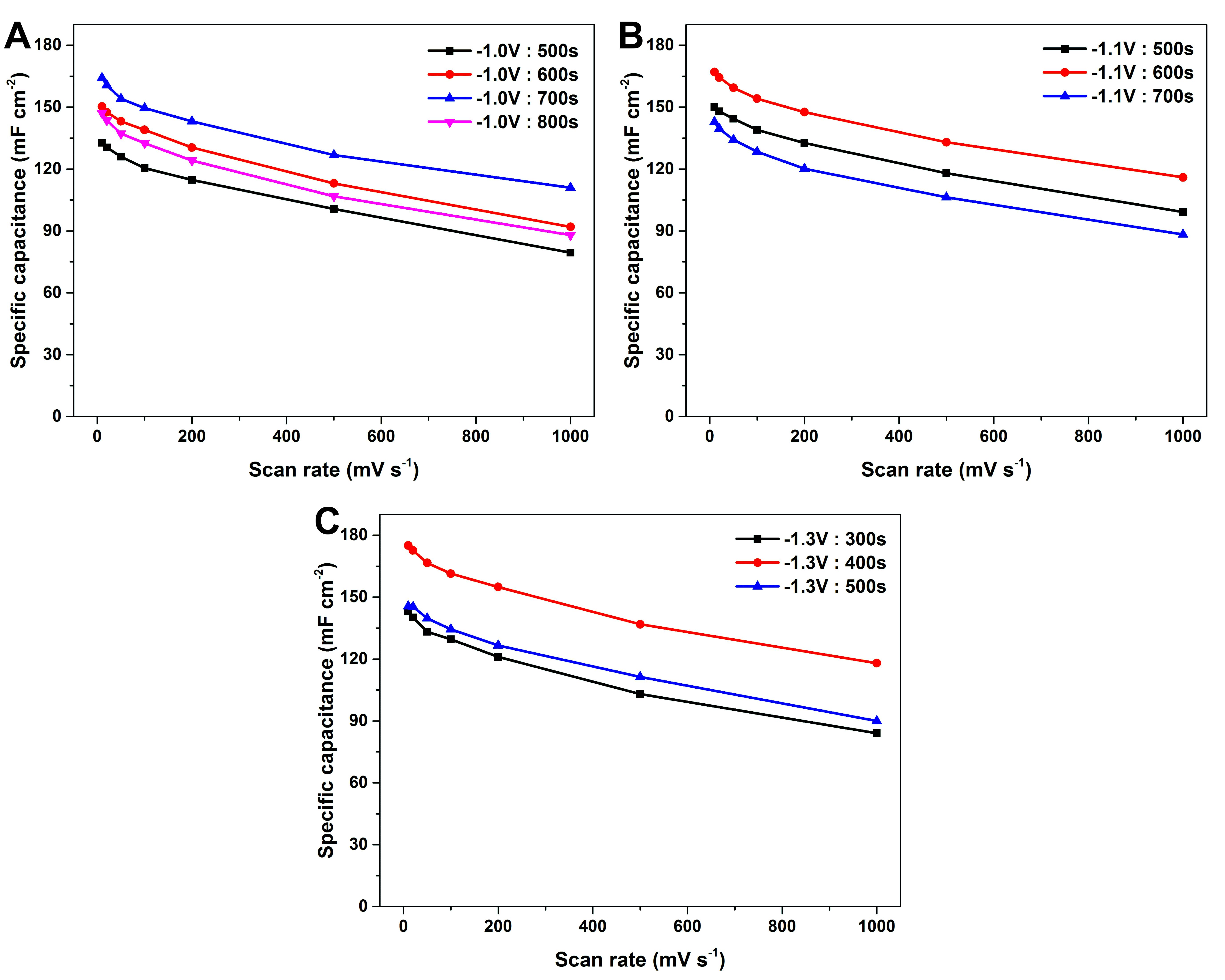


**Figure S6** Plots of specific capacitance of the electrodes prepared under deposition potentials of (A) -1.0, (B) -1.1, and (C) -1.3V with different deposition times versus scan rate.

1. Chen J, Sheng KX, Luo PH, Li C, Shi GQ**: Graphene Hydrogels Deposited in Nickel Foams for High-Rate Electrochemical Capacitor**s*. Advanced Material*s 2012**, 2**4:4569-4573.
